# Topological gene expression networks recapitulate brain anatomy and function

**DOI:** 10.1162/netn_a_00094

**Published:** 2019-07-01

**Authors:** Alice Patania, Pierluigi Selvaggi, Mattia Veronese, Ottavia Dipasquale, Paul Expert, Giovanni Petri

**Affiliations:** Network Science Institute, Indiana University, Bloomington, IN, USA; Department of Neuroimaging, Institute of Psychiatry, Psychology and Neuroscience, Kings College London, London, UK; Department of Neuroimaging, Institute of Psychiatry, Psychology and Neuroscience, Kings College London, London, UK; Department of Neuroimaging, Institute of Psychiatry, Psychology and Neuroscience, Kings College London, London, UK; Department of Neuroimaging, Institute of Psychiatry, Psychology and Neuroscience, Kings College London, London, UK; Department of Mathematics, Imperial College London, London, UK; EPSRC Centre for Mathematics of Precision Healthcare, Imperial College London, London, UK; Global Digital Health Unit, School of Public Health, Faculty of Medicine, Imperial College London, UK; ISI Foundation, Turin, Italy; ISI Global Science Foundation, New York, NY, USA

**Keywords:** Allen Human Brain Atlas, mRNA expression, Mapper, fMRI, Dopamine Topological Data Analysis

## Abstract

Understanding how gene expression translates to and affects human behavior is one of the ultimate goals of neuroscience. In this paper, we present a pipeline based on Mapper, a topological simplification tool, to analyze gene co-expression data. We first validate the method by reproducing key results from the literature on the Allen Human Brain Atlas and the correlations between resting-state fMRI and gene co-expression maps. We then analyze a dopamine-related gene set and find that co-expression networks produced by Mapper return a structure that matches the well-known anatomy of the dopaminergic pathway. Our results suggest that network based descriptions can be a powerful tool to explore the relationships between genetic pathways and their association with brain function and its perturbation due to illness and/or pharmacological challenges.

## Author Summary

In this paper, we described a gene co-expression analysis pipeline that produces networks that we show to be closely related to either brain function and to neurotransmitter pathways. Our results suggest that this pipeline could be developed into a platform enabling the exploration of the effects of physiological and pathological alterations to specific gene sets, including profiling drugs effects.

## INTRODUCTION

The human brain is a highly complex organ whose function emerges from the integration of cellular, anatomical, and functional circuits (Bassett & Gazzaniga, 2011). This complexity is thought to be crucial to provide the adaptability needed to maintain homeostasis and adapt to environmental changes. The architecture of the human brain is ultimately shaped by the human genome through the regulation of gene expression. In fact, the human brain consists of a set of differentiated regions each having a specific distribution of cell types and a microscopic and macroscopic anatomical organization that are the results of the differential expression of unique gene patterns during development that are kept stable when maturity is reached (Kang et al., 2011). Traditionally, genetic studies have investigated the association of genetic variants with a variety of brain disorders in large population studies (Ripke et al., 2014; Wray et al., 2018). However, these studies are not directly informative on the impact of these gene variants on brain structure and function. Imaging-genetic studies provided additional insights by exploring the effect of genetic variants and expression of gene sets on normal and pathological brains. They are, however, not without limitations (Bogdan et al., 2017); notably, they focus on the association between gene expression networks and brain phenotype in a limited number, or even single, brain regions, for example, the prefrontal cortex.

The availability of new datasets with vastly improved brain coverage and resolution, such as the Allen Human Brain Atlas data set (AHBA; Hawrylycz et al., 2012), offers a unique opportunity to explore the architecture of differential gene expression between brain regions. In fact, works that explored the intricacies of the human brain transcriptome are starting to uncover new knowledge about normal and pathological brain function. For example (Hawrylycz et al., 2015) revealed large transcriptional differences between brain regions. In addition, the same authors used a differential stability measure to isolate a set of genes whose patterns of differential expression across brain regions are highly consistent across donors, and showing that (i) differential expression patterns reflect the physical topography and the developmental trajectories of brain regions, (ii) these genes are strongly associated with different brain disorders, and (iii) the expression of these genes in the cortex is correlated with resting-state functional connectivity. Other authors found that resting-state functional connectivity networks are associated with between-brain-tissues correlated expression of a set of 136 genes (Richiardi et al., 2015). Taken together, these studies consolidate early evidence that mesoscale differences and similarities in structures and function across brain regions find their root in the architecture of the human transcriptome.

In particular, it has been shown that the brain global functional and structural networks are shaped by modules interconnected via “hubs,” balancing the integration and segregation of structures and functions, a hallmark of complex system that also optimizes a notion of brain economy (Bullmore & Sporns, 2009; Meunier et al., 2010; Simon, 1962; Sporns, 2013). Assuming that the cortex functional self-similarity (Expert et al., 2011; Tagliazucchi et al., 2013; Turkheimer et al., 2015) translates at the structural level, we expect this modular organization to be reflected in the brain transcriptome. Topological characteristics of the brain transcriptome have been already investigated by some authors (Kuncheva et al., 2017; Romero-Garcia et al., 2018) and related to function in animals (Wolf et al., 2011). However, a key limitation to the analysis of the topology of the brain transcriptomic is the high dimensionality of the data: the AHBA features the expression levels of more than 20,000 genes from six postmortem brains, profiled by ∼60,000 microarray probes in different brain regions that are spatially resolved at different level of anatomical coarseness (Hawrylycz et al., 2012). As a result, the analysis of the AHBA dataset poses a significant problem of dimensionality reduction that can be tackled by using different strategies in variable selection and feature extraction (Johnstone & Titterington, 2009).

Analysis methods based on network representation of brain function and structure have been extensively applied to a variety of imaging modalities, including magnetic resonance imaging, electroencephalography, magneto-encephalography, and diffusion tensor imaging. Here we present a new approach based on the Mapper algorithm (Singh et al., 2007) to reduce the dimensionality of microarray mRNA expression data from the AHBA while preserving topological information and characteristics of the human brain transcriptome. The Mapper algorithm has been developed to identify topological characteristics of datasets based on the distance between data points after applying a predefinite filter function (Singh et al., 2007). The algorithm constructs a series of networks describing the dataset at different levels of coarseness by grouping data points in nodes and connecting them according to their level of similarity. By using the composition of each node to map the samples to their anatomical location, we can represent the similarity of the genetic expression of samples obtained from different ROIs as network connectivity patterns. Mapper has been already successfully used to analyze high-dimensional behavioral, clinical, biological, and neuroimaging datasets (Jeitziner et al., 2017; Kyeong et al., 2015; Lum et al., 2013; Nicolau et al., 2011; Romano et al., 2014; Saggar et al., 2018).

In this work, we use Mapper as a method to relate gene expression and brain function and structure. We present three different applications of Mapper to the microarray AHBA data set: (i) the replication of the gene co-expression analysis originally presented by the Allen Institute for Brain Science (Hawrylycz et al., 2012); (ii) co-expression analysis of the gene list identified by Richiardi et al. (2015) that links gene co-expression and brain resting-state function; and (iii) topological co-expression analysis of the genes in the dopamine pathway. The two first case studies validate the pipeline by replicating previous findings, while the third focused on the characterization of the dopaminergic pathway from a spatio-genetic point of view. The dopamine system is crucially involved in many neuropsychiatric diseases (Martini et al., 2018; McCutcheon et al., 2019), and it has already been shown that coordinated expression of dopamine genes in the brain are associated with diseases and brain response to pharmacological challenges (Pergola et al., 2017; Selvaggi et al., 2019). Previous studies have suggested the existence of co-expression patterns across distinct structures of the brain in the case of neurotransmitter systems (Negi & Guda, 2017), but confirmatory evidence is still needed. Here, we show that different anatomical areas of the brain that are functionally connected by molecular pathways are also similar in terms of gene expression with a degree of similarity reflecting their position in the dopamine pathway.

## RESULTS

In this section, we briefly introduce the Mapper algorithm and the agreement matrix analysis we used, a detailed presentation can be found in the Methods section. We then present and discuss in detail the results we obtained on the three datasets we considered.

### Gene Mapper Networks and Agreement Matrices

The Mapper algorithm was first introduced in Singh et al. (2007) as a technique to extract low-dimensional skeletons for the classification of 3D shapes. In recent years, however, its usage as a data analytic tool has grown. In its simplest form, the algorithm takes a set of data points equipped with a similarity metric as input and returns a low-dimensional encoding of the backbone of the data that can be interpreted as a network. In the present study, we use a correlation-based distance to group together the gene expression vectors of brain tissue samples from the left hemisphere of six donors (Allen et al., 2012). To guide the algorithm with the local clustering, we use the first two principal components of the gene expression correlation matrix. Those two components explain between 21.6% and 30% of the variance, depending on the gene list considered. By varying the Mapper parameters, we obtain a series of networks describing the dataset at different levels of coarseness. We optimize the Mapper parameters to select the best networks in term of ratio between noise, that is, unconnected voxels, and signal, that is, connected components in the resulting network. Details about the construction and robustness are given in the Methods section and Supporting Information.

**Figure 1.**
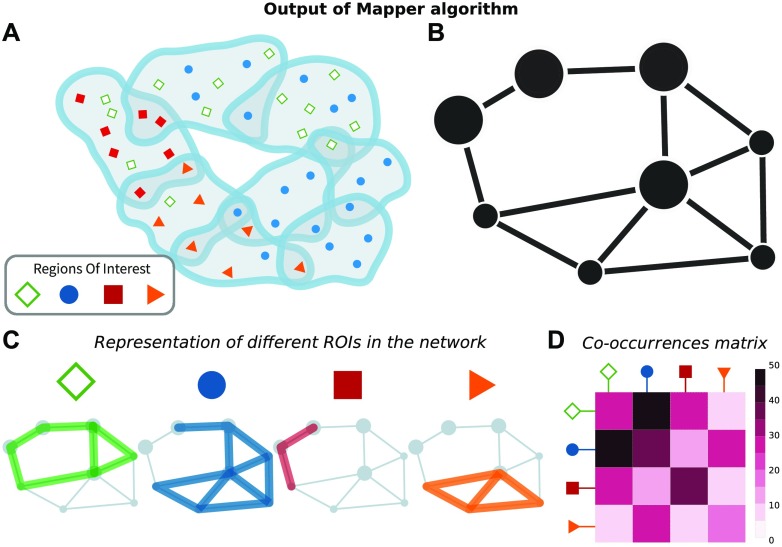
Representations and analytical tools for Mapper output. After slicing the data space in overlapping slices, partial clustering is applied to each slice (A). Since some data points belong to overlapping slices, they will belong to clusters in both slices (A). These overlapping clusters effectively produce a cover of the data space and can be summarized as nodes (containing the data points) linked to other nodes, whenever two clusters share data points (B). Data points correspond to samples of brain regions with specific anatomical and spatial characterizations. It is therefore possible to investigate how distributed or localized the samples of a certain Region of Interest are (C). This information is succinctly described by the co-occurrences matrix that counts how often nodes belonging to different ROIs belong to the same Mapper node (D).

Each node in the network produced by Mapper represents a cluster composed by samples with very similar gene expression profiles and similar loading on the first and second principal components. Therefore we can represent the similarity of the genetic expression of samples obtained from different regions of interest (ROIs) by mapping the samples constituting each node to map the samples to their anatomical location. The connectivity patterns between nodes in the network further highlight this similarity. In particular, we can study the subgraphs determined by nodes that contain samples from the same ROIs. Scattered subgraphs indicate a loose similarity within the specific ROI and a higher similarity with other ROIs; see Figure 1 for an illustration. Given a set of parameters {*σ*} for the Mapper, see Methods for details, this information can be further summarized using a co-occurrence matrix *A*[*σ*], where each element *A*_*ij*_[*σ*] counts the numbers of times a sample from region *i* and a sample from region *j* are mapped to the same node. As the network produced by Mapper depends on the parameters {*σ*}, we introduce an agreement matrix to summarize the information gained from Mapper at different points in the parameter space. For each optimal set of parameter, we obtain a co-occurrences matrix and compress the information obtained from the networks corresponding to different parameter choices in an agreement matrix *A* = 〈*A*[*σ*]〉_*σ*_, obtained by averaging across all matrices and retaining the nonzero elements that have at least one connection in all the networks. Throughout the paper, when we compare the information gathered from Mapper with other data sources that can also have a matrix summary, we always use the agreement matrix built considering all optimal parameters as the Mapper representative.

### Data Sets

We applied the pipeline described in the previous section to three different sets of genes: the whole human genome (∼29,000 genes), a list of 136 genes that support synchronous activity in brain networks as shown by Richiardi et al. (2015), and a list of 56 genes related to the dopamine system. The dopamine list was created by interrogating the Gene Ontology database (http://geneontology.org) and the Panther gene classification system (Mi et al., 2013) from which the “Dopamine receptor mediated signaling pathway (P05912)” list was selected. The gene expression data for all three gene lists we considered come from the microarray data of the Allen Human Brain Atlas (http://human.brain-map.org). The original log2 data were transformed into z-scores following the methodology present in Rizzo et al. (2016). We use the first dataset to validate the proposed methods within a fully gene-focused setup. The analysis of the second dataset instead highlights the capacity of this method to meaningfully link the genetics and activation level of specific ROIs. Finally, the dopamine system analysis provides new insights in brain organization. We used both the agreement matrices and standard network properties, for example, shortest path distances, to compare our results with previous analysis done on the same dataset. Functional connectivity matrix was computed using high-resolution resting-state fMRI data from 20 subjects (5F/5M aged 26–30; 5F/5M aged 31–35) randomly selected as part of the Human Connectome Project (h2ps://db.humanconnectome.org/). Please find full details about data acquisition and preprocessing in Supporting Information.

### Validation against the Allen Brain Atlas

**Figure 2.**
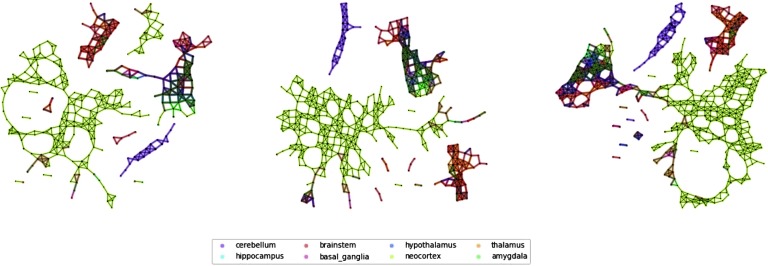
Mapper networks of the Allen Brain Atlas dataset. From left to right, we show some Mapper networks obtained for the same window size but different overlaps between windows (overlap 25%, 30%, 35%). The networks display very similar qualitative properties, for example, the separation of the cerebellar areas, which we further characterize using the agreement matrices.

Figure 2 shows a some examples of Mapper networks (parameters window size 5%, overlap = 20%, 25%, 35%; see Methods) obtained from the Allen Human Brain Atlas (AHBA) dataset at different levels of coarseness. We can see that in all cases a few major connected components in the network: a small one, containing only elements of the cerebellum, a giant component containing mostly cortical samples, and smaller subcortical components. As the overlaps increase, we can see how the larger components start developing connections toward the components containing samples from subcortical ROIs, while the component containing samples from the cerebellar cortex is still very much isolated in all cases.

**Figure 3.**
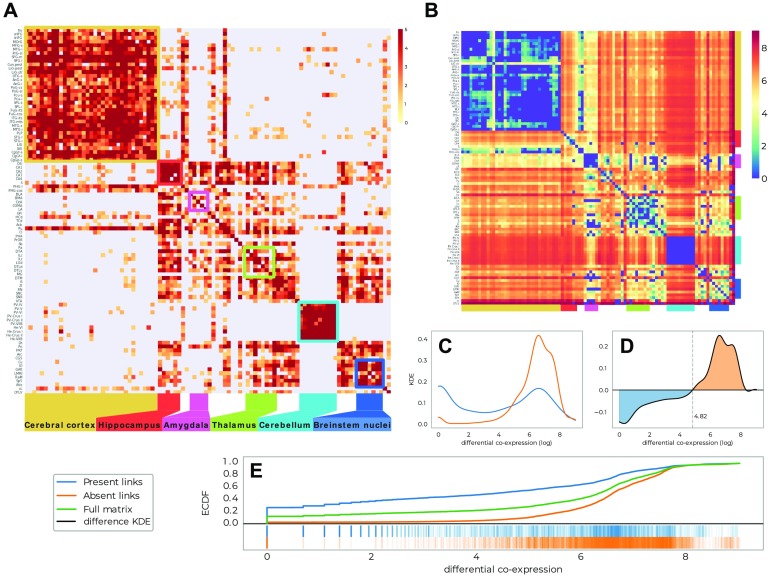
Comparison of the output network with differential analysis from the Allen Brain Institute. (A) Agreement matrix for Mapper co-occurrence matrices for parameters (window size, overlap) = {(5, 25), (5, 30), (5, 35), (6, 20)}, see Supporting Information for more information on the parameter choice. (B) Differential gene expression matrix, reproduced from Hawrylycz et al. (2015). (C) Distributions of (log) differential gene expression for links that are and are not present in the Mapper networks. (D) Difference between the distributions in C). (E) Cumulative distributions.

Although some of these patterns and their stability can be guessed by direct observation, the distinction is much clearer when we consider the structure of the agreement matrix in Figure 3A (for parameters (window size, overlap) = {(5, 25), (5, 30), (5, 35), (6, 20)}; see Supporting Information for more information on the parameter choice, where we can clearly distinguish four blocks representing: the cerebral cortex, the hippocampus, the cerebellum, and brainstem nuclei. We can identify regions of the cerebral cortex that have higher connectivity with the two blocks from the subcortex, indicating the connection in the network of the central community to the peripheral structures. We are now in a position to compare the findings of Mapper to the original paper of (Hawrylycz et al., 2015).

In their work, Hawrylycz et al. present their findings using a consensus map of all genes differentially expressed between any pair of 96 regions in at least five of the six total donors. The matrix they present in Figure 1A (Hawrylycz et al., 2015) counts the number of genes with at least a fold change > 3 in expression level between each structure pair. We compare the agreement matrices that represents the connectivity of all the considered Mapper networks with the co-expression matrix of the original paper, which is summarized in Figure 3. To guide the eye, we ordered the regions in Figure 3 in the same order as in Hawrylycz et al. (2015, Figure 3B) and it is easy to see that the Mapper agreement matrix reproduces the general structure of the differential gene expression matrix. In order to quantify this effect, we delve a little deeper in the relationship between the links of the Mapper networks and the standard differential gene expression techniques. In fact, we show that Mapper extracts the connections with lower differential of gene expression. We compare the differential gene expression between the ROIs that are connected in Mapper and the ones that are not. In particular, we expect that the links present should be characterized by smaller differential expressions, since the presence of a link suggests a higher similarity between the samples contained in the nodes, whereas the absence of a link supports the opposite. To quantify this difference we use the Kolmogorov–Smirnov statistic which measures a distance between the empirical distribution functions of two samples (Figure 3E). We find that links present in Mapper generated networks are up to 27% more likely to include low differential co-expression between the ROIs than the complete full matrix, and up to 45% more likely than the ones ignored by the algorithm construction (Figure 3C). When confronting the two differential co-expression distributions for present and absent links (Figure 3D), it is clear that Mapper tends to contain more links with a differential gene expression of at most *e*^4.85^ than the ones it excludes.

**Figure 4.**
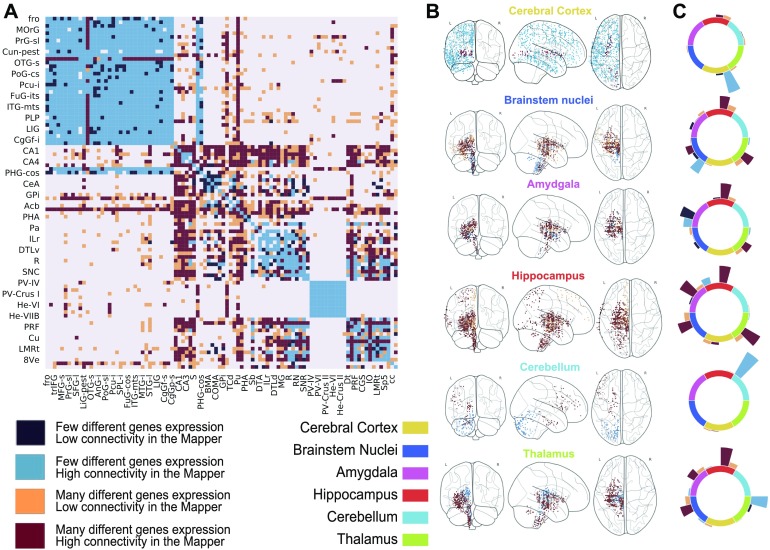
Comparison between the connectivity and the differential gene expression for links present in the Mapper network. (A) The agreement matrix colored according to four regimes based on the combinations of sparse/dense connectivity in the Mapper network (defined as more or less than 2 co-occurences between the regions) and of low/high differential gene expression (defined using the trade-off detected in Figure 3D). (B) and (C) Visualization of the predominant regimes of the links connecting each ROI with the rest of the hemisphere. In B, only samples belonging to areas connected with the ROI are shown coloured according to their relative regime. In C we show the total number of links between the ROIs belonging to each regime.

In addition to the large-scale results reported above, it is possible to focus on the comparison between the brain systems analyzed in Hawrylycz et al. (2015). In Figure 4A, we reproduce the agreement matrix colored according to four regimes. These are based on the combinations of sparse/dense connectivity in the Mapper network and of low/high differential gene expression in the nodes. A link is qualified as dense if there are on average more than two co-occurrences between the nodes, and the low/high differential expression is defined by the trade-off detected in Figure 3D. It is clear that the two most represented cases are as expected: few different gene expressions and dense Mapper connectivity, in light blue, versus inconsistent genes expression profiles and sparse network connectivity, in dark red. We give more details in the Supporting Information about how the classification in four regimes is done.

Beyond this broad classification in four regimes, there are more subtle connectivity patterns: for example, the cerebral cortex and the cerebellum are clearly more homogeneous than the hippocampus or the amygdala (Figure 4B). To extract all the information from the Mapper results and interpret them correctly, we need to go further. We can quantify for each ROI how many links of a certain type link to other ROIs. We summarize this construction in the circular bar plots of Figure 4C. In general, we can see how for low differential gene expression Mapper tends to always be densely connected, whereas the converse is not always true with clear examples in the hippocampus and amygdala.

Using this information, we can compare the Mapper results with those of the AHBA for each system/ROI:

•  **Cerebral Cortex:** The cerebral cortex appears to be very connected in the Mapper network, consistent with the idea that the basic architecture across the entire cortex is similar or “canonical.” The presence of both low differential genes expressed and high connectivity is indicated by the light blue shading comprising cortical gyri ordered from the frontal pole (fro) to cingulate gyrus (CgGP-s). The sole exception is the visual cortex LiG-str, which had a uniquely diverse gene-expression that the network was not able to discern.
•  **Cerebellum:** The same connectivity pattern can be seen for the cerebellum. Hawrylycz et al. (2015) noticed how the internal homogeneity across subdivisions of the cerebellum, with samples from different cerebellar lobes listed from “PV-IV” through “He-VIIB” show no internally differentially expressed genes. This peculiarity is clearly indicated in the Mapper outputs, as the cerebellum always creates a connected component of its own in the network.
•  **Hippocampus:** In Hawrylycz et al. (2015), the hippocampus showed a distinct pattern of gene expression across its highly distinct and stereotyped anatomical divisions. This peculiar pattern of differential gene expression is not reflected in the Mapper generated network by a disconnected component. Instead, the samples from the various hippocampal structures are highly connected within the subcortex and loosely connected with parts of the cortex (LiG-pest, SPL-s, SPL-i, STG-l). This pattern is different from its close relative, the cortex, and even more distinct from that of evolutionary older brain regions.
•  **Amygdala:** In their work, Hawrylycz et al. found that the amygdala are very similar to one another while very different from other brain regions, in the Mapper network these regions are instead loosely connected within the subcortex and within themselves.
•  **Thalamus, Brainstem Nuclei:** The thalamus and brainstem nuclei show a great deal of complexity in the differential gene expression matrix. This is confirmed in the Mapper agreement matrix. We can see how these two structures are interconnected in the network but with high proportion of low connectivity links, suggesting a less clear organization than the cerebral cortex and cerebellum.

### Across Modalities Validation—The Richiardi List

**Figure 5.**
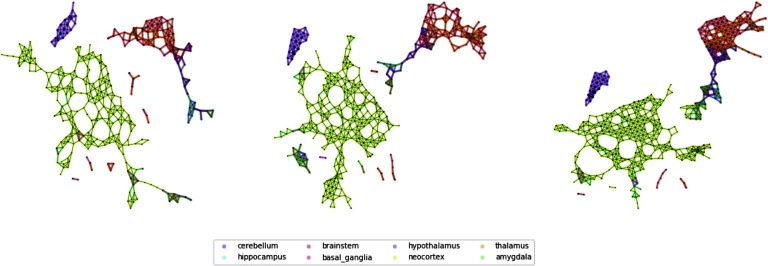
Mapper networks on reduced Richiardi functional list. From left to right, we show some Mapper networks obtained for different overlaps between bins and same window size (overlap 25, 30, 35). The networks displays the separation of the cerebellum and two larger components, composed by cortical and subcortical areas respectively, which we further characterize using the agreement matrices.

Richiardi et al. (2015) identified a list of 136 genes whose expression is correlated with the so-called resting-state functional connectivity. We recomputed the Mapper network using the gene list identified by Richiardi et al. (2015) instead of the whole genome. We then compared this Mapper network to the functional connectivity matrix derived from resting state fMRI data from the Human Connectome Project. In the Mapper generated networks we observe that while the cerebellum again constitutes a stand-alone component, the remaining samples are divided in two larger and anatomically coherent components: one containing most of the cortical samples and the other one containing mainly subcortical ones (see Figure 5).

**Figure 6.**
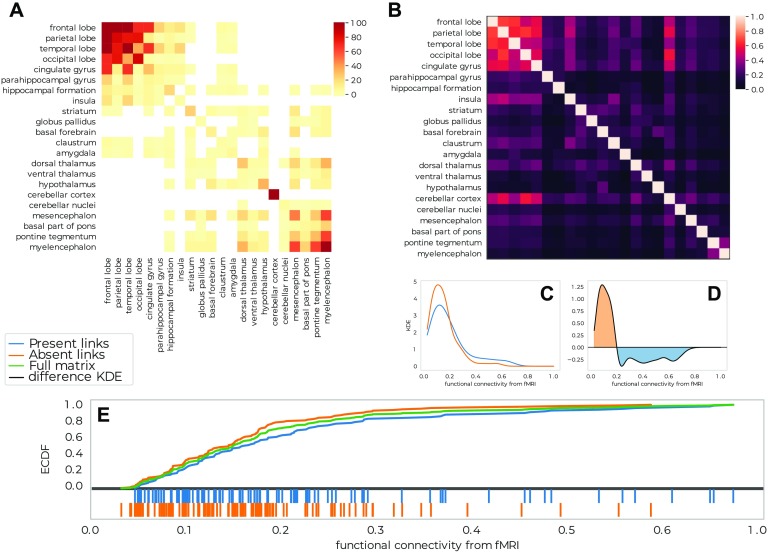
Comparison of the Mapper network with an average functional network from resting-state fMRI. (A) Agreement matrix for Mapper co-occurrence matrices for Mapper networks built using the Richiardi list. (B) Average fMRI synchronization between regions. (C) Distributions of fMRI correlations for links that are present or absent in the Mapper networks. (D) Difference between the distributions in C. (E) Cumulative distributions.

In this case study we compare the properties of the Mapper links to the functional connectivity values and find that the Mapper links correlate with higher functional connectivity. The links extracted by the Mapper generated network are up to 14% more likely to include high functional connectivity between the ROIs than the complete full matrix, and up to 29% more likely than the ones ignored by in the construction of the Mapper network. These values were computed using the Kolmogorov-Smirnov statistic measuring the highest gap in the cumulative distributions (see Figure 6E). Comparing more closely the distributions of functional connectivity for links presents and absent in the Mapper network, we can identify the level at which the change in trade-off occurs (Figure 6D).

In order to remove highly differential outliers, Richiardi et al. (2015) decided to focus their analysis on 1,777 cortex samples mappable via their Montreal Neurological Institute (MNI) coordinates to 13 functional networks (see Supporting Information for full list) excluding 1926 samples from the basal ganglia, cerebellum, and deep gray matter regions including the hippocampus.

**Figure 7.**
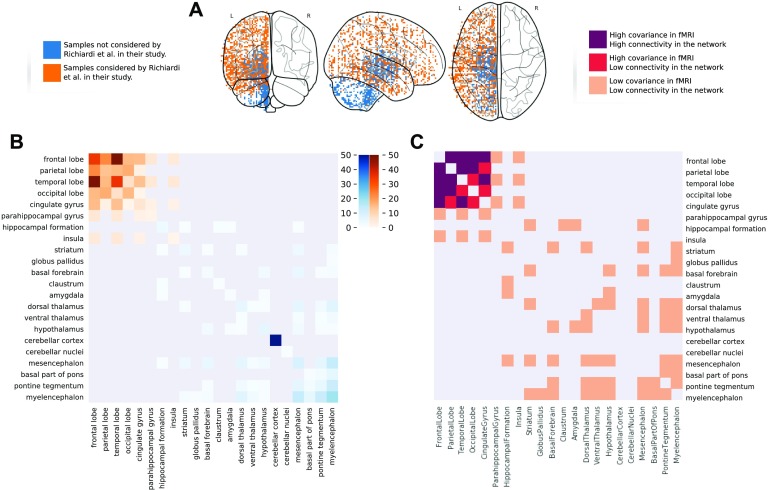
Comparison of the connectivity for the samples correlated with synchronous activity in fMRI and the rest of the brain. (A) A visualization in the MNI space of the samples considered by Richiardi et al. (2015) (in orange) and the samples excluded in their work (in blue). (B) The reduced Mapper agreement matrix containing only the co-occurrences within the samples considered by Richiardi et al. (in shades of orange) and the rest of the hemisphere (in shades of blue). (C) The agreement matrix colored according to three regimes based on the combinations of sparse/dense connectivity in the Mapper and low/high average functional connectivity.

As with the analysis of the AHBA whole genome, we can use this information to define different regimes for the links and compare in more detail the connectivity in Mapper for the samples considered by Richiardi et al. (2015) in their analysis and those ignored. The results are presented in Figure 7. When considering the connectivity of the ROIs in the Mapper network via the co-occurrence matrix we can see a similar pattern to the one found in the fMRI covariance matrix where the ROIs involved in the functional networks were studied by Richiardi. All the samples not considered by Richiardi in their work tend to not be clustered together in the Mapper network. Vice versa, the samples that Richiardi found to correlate with fMRI tend to be clustered together in the network, with the exception of the temporal, occipital lobes and cingulate gyrus, which are less densely connected.

### Dopamine System

**Figure 8.**
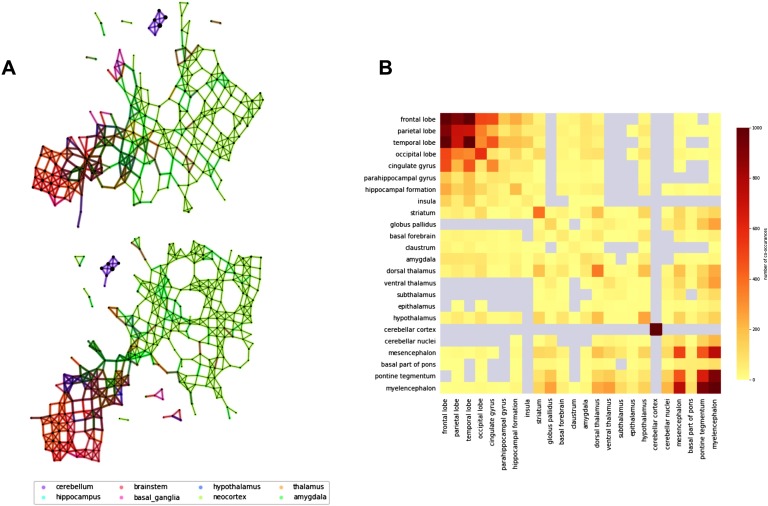
Mapper networks of the dopamine system. (A) Agreement matrix for Mapper co-occurence matrices for parameters (window size, overlap) = {(5, 25), (5, 30), (5, 35), (6, 20)}. From top to bottom, we show some Mapper networks obtained for (window size, overlap) = (5, 25) - top, and (window size, overlap) = (6, 20) - bottom. (B) The networks display very similar qualitative properties, such as the separation of the cerebellar areas, which are further characterized using the agreement matrix.

We can see two main connected components in the Mapper network (Figure 8A): a small component containing only elements of the cerebellum and a giant component containing most of the samples. In the giant component two anatomically coherent modules are easily distinguishable: one containing samples from the cortex, the other one from the subcortical ROIs.

**Figure 9.**
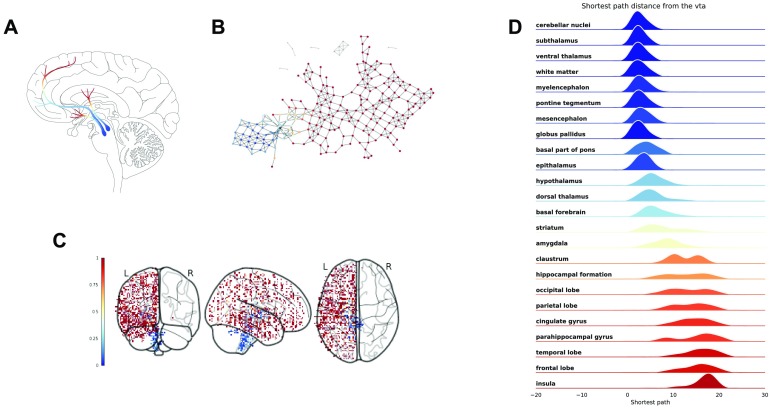
Distribution of shortest path distance in the output networks. For each network we identified the nodes in the network containing samples from the ventral tegmental area and computed the shortest path distance from these nodes to the rest of the network. (A) The dopaminergic pathway (thanks to Gill Brown, London College of Communication, UK). (B) Each node/cluster is colored according to its average path distance from nodes containing samples from the ventral tegmental area. (C) The samples from the left hemisphere in the MNI space colored according to the average path distance of the nodes they belong to in the network. (D) The distribution of path distance values for each ROI ordered (top to bottom) according to their mean value from closest to farthest.

From the agreement matrix we can clearly distinguish the two modules as deep red blocks (Figure 8B). The fact that a significant portion of the high connectivity is between ROIs indicates that the clusters in the network are very inhomogeneous in their composition, that is, it is very likely to find samples from different ROIs clustered together. The organization of the network in two modules is reminiscent of the anatomical organization of the mesolimbic dopamine pathway characterized by a crosstalk between cortical and subcortical structures. For this reason we decided to study the organization of the network relative to nodes containing samples from the substantia nigra and ventral tegmental area (VTA). These regions were chosen because they are the areas of the brain most densely populated by dopamine-producing neurons and therefore thought to be the starting point of the dopaminergic pathway in the brain (Nair-Roberts et al., 2008; see Figure 9A). We then calculated the shortest path distance from these nodes to every other node in the main connected component. We discuss here the results for seeds chosen in the ventral tegmental area, but the analysis is consistent for the substantia nigra (see Figure SI.4 for a comparison of the results between the two ROIs Supporting Information.

Each node/cluster thus had a value assigned to it that represents its shortest distance in the Mapper network from the ventral tegmental area (Figure 9B). Since each sample in the left hemisphere belongs to one or more nodes/clusters in the network, the shortest path distance values was mapped from the nodes to the samples (Figure 9C). We then studied the distribution of these values inside each ROIs (Figure 9D). In blue the ROIs contain samples in the same cluster or closer to the substantia nigra. The samples from these ROIs are mostly contained in the same network module. The ROIs are ordered from top to bottom from closest to farthest. In lighter blue and oranges are the ROIs with samples in the part of the network bridging the two modules. In red is the other module which encompasses most of the cortex. Interestingly, the ROIs in lighter blue and orange (e.g., the striatum and the thalamus) are actually ROIs that contain a high number of synapses between axons coming from the brainstem and neurons projecting into the cortex (Parent & Hazrati, 1995). These results show that indeed the Mapper network reproduces closely the anatomical structure of the dopaminergic pathway.

## DISCUSSION

Dimensionality reduction methods aim at capturing salient characteristics of datasets and are thus essential tools to study complex and complicated systems. However, what is gained in size is often lost in interpretability; it is therefore crucial to find lower dimensional representations of high-dimensional spaces that are easily associated to other measurements or observables of the system under study. Among topological simplification tools (Edelsbrunner & Harer, 2008; Petri et al., 2014), Mapper is the method of choice to achieve this goal. Although a powerful tool that yielded significant results (Lum et al., 2013; Nicolau et al., 2011; Saggar et al., 2018), little work has focused on the cross-validation of its results across modalities and datasets. In this study, we built a Mapper pipeline designed to extract pattern of similarity in genetic co-expression and crossed examined them through brain anatomy, with the aim to link genetic expression to neuroscience. We show that Mapper is an efficient topological simplification tool that is able to extract meaningful patterns of gene co-expression that are related to brain function and structure.

First, we validated the pipeline by replicating most of the co-expression patterns obtained for the Allen Human Brain Atlas (AHBA) in Hawrylycz et al. (2015, 2012). We found that in most cases the Mapper algorithm creates densely connected areas between samples that have a low differential gene expression such as areas within the neocortex. These findings matched previous results (Hawrylycz et al., 2015) obtained with other analysis techniques such as Weighted Gene Co-expression Analysis (WGCNA) (Zhang & Horvath, 2005). However, the opposite does not hold for other areas such as the hippocampus and the amygdala. Clear outliers are samples from the amygdala that even with low differential expression tends to be overall sparsely connected. A possible explanation could be in the loadings of the first two principal components on these areas, which informs the slicing into bins for the local clustering. If the samples from the amygdala do not end up in neighboring bins they will not cluster together. Indeed, a potential solution would be to adopt underlying filters based on recent dimensional reduction techniques designed to reduce the effects of heterogeneous sampling of gene space (McInnes & Healy, 2018).

We were also able to reproduce the results obtained in Richiardi et al. (2015) by correlating resting-state fMRI connectivity patterns with gene co-expression from a curated list of genes. This is particularly interesting as no specific choice was required to extract a topological manifold, the Mapper network, that linked directly gene expression to function. Moreover, recent results showed that the landscape of resting and task brain activations can be well approximated using Mapper on voxel-level activation data (Saggar et al., 2018). The homogeneity of the methods and descriptive spatial scales would then naturally allow to fuse the two approaches by characterizing the activity clusters at the genetic level and vice versa, or by producing brain activity Mappers informed by the underlying gene expression Mapper networks.

Equipped with these validations, we turned to a subset of genes associated with a crucial neurotransmitter: dopamine. Remarkably, the Mapper network follows closely the anatomical dopaminergic pathway, elegantly relating complex genetic co-expression patterns to their physical manifestation. Indeed, the analysis of the co-expression of dopamine genes was able to extract biologically meaningful features that have not been detected previously (Negi & Guda, 2017). In particular we detected a network that is composed of two main interconnected modules: one containing cortical samples and the other containing subcortical regions, in line with previous reported differences in gene expression between cortical and subcortical regions (Hawrylycz et al., 2015). Moreover, our results also indicate that cortical and subcortical gene expression are not totally independent but share similarities. This is of particular interest since dopamine is one of the key neurotransmitters involved in the modulation of the cross-talk within the cortico-basal ganglia-thalamo-cortical loop (CBGTC) (Haber, 2016; Peters et al., 2016). The structures identified by Mapper suggest that the anatomical and functional organizations of the CBGTC are reflected, or even possibly driven, by coordinated expression of dopamine genes in the loop. Results from the shortest path distance analysis offer further support for this interpretation. They show a gradient of mRNA expression of dopamine genes from the subcortical seed regions, the VTA and the substantia nigra, to cortical ROIs (in red in Figure 9). The cortical ROIs are the furthest in terms of gene expression similarities in the Mapper generated network, whereas ROIs within the basal ganglia and the thalamus (light blue and orange in Figure 9) were closer to the seeds. Interestingly, dopaminergic projections from the VTA and the substantia nigra to the basal ganglia play a crucial modulatory role in the CBGTC loop and have been implicated in the pathophysiology of different neuropsychiatric conditions (Martini et al., 2018; McCutcheon et al., 2019).

The results presented here suggest that Mapper can be reliably used to test hypotheses regarding the implication of specific genes or set of genes in brain function by producing associated networks and their relation with other imaging modalities such as EEG, MEG, fMRI, and potentially generic combinations of different modalities. Moreover, the preprocessing of the data input to Mapper is minimal compared with traditional genetic studies (Hawrylycz et al., 2015; Krienen et al., 2016; Richiardi et al., 2015), making it easier to follow the effect of each ingredient on the results, and the results are stable across large parameter ranges see Supporting Information; and previous results (Carriere et al., 2018; Dey et al., 2016). This point is of particular importance since the field is starting to raise concerns about reproducibility issues related with data preprocessing (Arnatkeviciut, Fulcher, & Fornito, 2019).

Mapper uses gene expression similarity to build a network structure where higher size translates to highly co-expressed brain samples. This technique could be further developed to study the effect of the overlap and window size parameters on the density of the resulting networks. The study of other topological structures, like cycles and holes (Cámara, 2017; Carlsson, 2009; DeWoskin et al., 2010) and their evolution across different scales could provide interesting insights on the involvement of gene expression onto the organization of brain function at a local level.

To summarize our main contribution, Mapper returned a network structure of co-expression of genes related to the dopaminergic pathway that is largely supported by current knowledge about the physiology of the dopaminergic pathway in the brain, and suggests new insights into the functioning of the CBGTC loop. The results call for further investigations in disease populations to test whether the structure identified here is altered. This could provide an integrated representation of the spatial, genetic, and functional architecture of the brain, leading to direct applications in understanding the interactions and effect between neurotransmitters and finally sheding light on mechanisms underlying (i) mental health disorders and (ii) the effects and side-effects of their associated treatments.

## METHODS

To study the hidden organization of the AHBA gene expression data set, we use the Mapper algorithm (Singh et al., 2007). The Mapper algorithm builds a low-dimensional skeleton of the dataset by using similarity information intrinsic to the original data guided by other well-established low-dimensional embedding techniques. In our work we set out to construct a skeleton that would represent the correlation similarity between the genetic expression of different brain regions.

### Mapper

**Figure 10.**
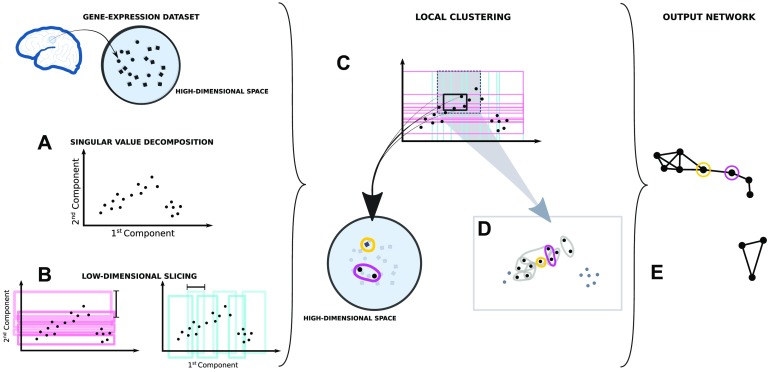
Description of the Mapper algorithm. Each point in the dataset is considered as a vector of gene expression in a high-dimensional space (∼29,000 dim, 136 dim, or 56 dim according to the number of genes considered). (A) The first two principal components of the gene covariance matrix are computed reducing the initial dimensions to 2, (B) then the data set is in overlapping windows of equal density along both components. (C) The information from both slices is used together to divide the dataset in overlapping rectangular bins. This way, each bin will have samples having similar weight in both component. (D) The samples from each bin are clustered independently from each other according to the gene expression correlation between the samples in the original high-dimensional space. (E) The information is then summarized in a network where each independent cluster is represented by a node. In the network two nodes are connected if the relative clusters belong to neighboring bins and share samples.

The algorithm requires many parameters and choices that help build the network that best describes the aspect of the dataset one wants to highlight. One can summarize the algorithm in four major parts (see Figure 10 for a detailed description):

#### Low-dimensional embedding

A set of features of the dataset are chosen as a local guide to the slicing process. In our case we chose the first two components of the singular value decomposition of the samples’ gene expression covariance matrix.

#### Low-dimensional slicing

Each dimension of the embedding in Figure 10A is considered separately. The dataset is divided using overlapping windows. The size of the windows and their overlap are key parameters for the local topology of the output network.

#### Local clustering

Using the combined information of the slicing in Figure 10B, the dataset is divided in overlapping bins. A clustering algorithm is run in parallel on each bin independently. We chose to run a density-based clustering algorithm (Campello et al., 2015) with a correlation-based similarity as distance.

#### Building the network

Each cluster found in Figure 10C is represented by a node in the network. Samples contained in the overlap between windows will be present in more than one cluster. An edge is drawn between nodes that share samples to represent the nonempty intersection.

The only two free parameters in our application of the algorithm are the window size and overlap at step in Figure 10B. In general, increasing the window size will lead to a Mapper network with fewer nodes, whereas increasing the overlap will lead to a denser network. At one extreme, with fixed window size and no overlap, the resulting network will be formed of isolated nodes with no edges. At the other extreme, for overlap close to 100% of the window size, we will have a densely connected lattice.

The way the changes in window overlap influences the connectivity in the output network mirrors the way gene expression similarity shapes structure and function at different scales. In order to study this effect, we decide not to choose a single overlap value. Instead, we use basic network features, number of connected components, and edge density, to get a set of optimal parameters and obtain a series of network descriptors at different level of coarseness. In the Supporting Information we show the optimal parameters for each list of genes.

#### Agreement matrix

For each network, we can analyze the gene expression similarity within anatomical brain regions via the study of node connectivity in the network. To summarize the anatomical information stored in each network connectivity we build a co-occurrences matrix, where each element *C*_*ij*_ of the matrix represents the number of times that two samples from ROI *i* and ROI *j* are mapped in the same node/local cluster.

We condense the connectivity information from the networks built using all the optimal parameters in a unique matrix, where each element is the average co-occurrence across all networks and *C*_*ij*_ is nonzero only if ROI *i* and *j* are connected in all the networks.

It is useful to notice that the co-occurrence matrix can also be constructed considering only a subset of the samples present in the network. This approach gives a more selective account of the correlation between anatomical regions, where the influence of the ignored regions is still accounted for by the network but ignored in the numeric computation of the matrix. This effect can be noticed in Figure 7 where the connectivity of the samples considered by Richiardi et al. (2015) in their work that was studied separately from the rest of the samples in the Allen Human Brain Atlas.

### Code Availability

The code used for the analysis showed in this paper can be found at the following repository: https://github.com/alpatania/AHBA_microarray_Mapper/

## AUTHOR CONTRIBUTIONS

Alice Patania: Conceptualization; Formal analysis; Investigation; Methodology; Software; Validation; Visualization; Writing – Review & Editing. Pierluigi Selvaggi: Conceptualization; Data curation; Validation; Writing – Review & Editing. Mattia Veronese: Conceptualization; Data curation; Validation; Writing – Review & Editing. Ottavia Dipasquale: Data curation. Paul Expert: Conceptualization; Data curation; Project administration; Writing – Review & Editing. Giovanni Petri: Conceptualization; Supervision; Writing – Review & Editing.

## FUNDING INFORMATION

Paule Expert, Imperial NIHR Biomedical Research Centre: NIHR-BRC-P68711. Giovanni Petri, Compagnia di San Paolo (http://dx.doi.org/10.13039/100007388), Award ID: ADnD Grant. IntesaSanpaolo Innovation Center. The funder had no role in study design, data collection, and analysis, decision to publish, or preparation of the manuscript. Pierluigi Selvaggi, NIHR-BRC at South London and Maudsley NHS Foundation Trust and King’s College London (http://dx.doi.org/10.13039/100013376). Mattia Veronese, NIHR-BRC at South London and Maudsley NHS Foundation Trust and King’s College London (http://dx.doi.org/10.13039/100013376). Ottavia Dipasquale, NIHR-BRC at South London and Maudsley NHS Foundation Trust and King’s College London (http://dx.doi.org/10.13039/100013376). Paul Expert, EPSRC award EP/N014529/1 funding the EPSRC Centre for Mathematics of Precision Healthcare at Imperial.

## TECHNICAL TERMS

Resting state: An fMRI acquisition during which the subject is awake and conscious but is not stimulated nor performing any task.
Topology: A branch of mathematics that describes spaces in relation to the properties of their shape.
Filter: A mapping that associates a real number to every data point in the dataset.
Dopaminergic pathway: A set of four neuronal pathways in the brain that synthesize and release the neurotransmitter dopamine.
Similarity metric: A nonnegative function such that its value is greater when two points are closer to each other.
Principal components: A set of linearly uncorrelated variables that each account for a given amount of the variability of the dataset, mostly used for exploratory analysis.
Cluster: A group of data points that are more similar to each other than to the rest of the dataset.
Subgraph: A graph induced by a subset of vertices with the edges connecting them.
Shortest path distance: A distance between two vertices of a graph defined as the minimum number of edges that connect them.
Edge density: The ratio between the number of edges present in the graph and the number of all possible ones.
