# Supplementary material for: Topological gene expression networks recapitulate brain anatomy and function
Source: Netw Neurosci. 2019 Jul 1;3(3):744–62. doi: 10.1162/netn_a_00094 (PMC6663211; doi:10.1162/netn_a_00094)
Supplement: Supplementary file 1 [file netn-03-744-s001.pdf]

Patania, A., Selvaggi, P., Veronese, M., Dipasquale, O., Expert, P., & Petri, G. (2019). Supporting information for "Topological gene expression networks recapitulate brain anatomy and function." *Network Neuroscience, 3*(3), 744–762. https://doi.org/10.1162/netn_a_00094

Topological gene-expression networks recapitulate brain anatomy and function

Alice Patania, Pierluigi Selvaggi, Mattia Veronese, Ottavia Dipasquale, Paul Expert and Giovanni Petri

Supporting Information

1 Optimization of parameters for the Mapper algorithm

The Mapper method has 5 parameters that one needs to fix before running the algorithm:

filter the filter is a map, or series of maps, which will guide the subdivision of the data-set in overlapping bins. We decided to use the first 2 principal components of the gene-expression covariance for the each list of genes.

coarseness the level of coarseness in the output network (number of nodes, threshold of connectivity) is determined by the size of the overlapping bins on which the clustering algorithm is performed. Large bins thus give fewer nodes than smaller ones. Bins with a high percentage of overlap will give a densely connected network with many nodes. Vice-versa, lower overlap will give more disconnected components with few edges.

- **window size** is the size of the overlapping windows used to slice each principal component independently,
- **overlap** is the overlap between the windows used to slice each principal component independently.

clustering A clustering algorithm is run independently on each bin. The similarity measure used by this algorithm is the key to the interpretation of the output network.

- **clustering algorithm** We chose HDBSCAN Campello et al. [2015] to use a density based hierarchical algorithm that has a less supervised cutoff;
- **similarity measure** Considering that we intended to study the relationship between gene-expressions, we chose $1-$correlation between the gene-expression vectors.

Finding the window sizes that give the most reasonable average bin size

We look at 50 different window sizes, from 1 to 50. The size is chosen so that a fix percentage of the data points are present in each window. To reduce the number of networks to build, we reduce the number of window sizes in consideration by studying the resulting

average bin size. In this preliminary study the windows are built with no overlap. After intersecting the two sliced data-set, we obtain the list of all bins to consider for clustering. We compute the average bin size for each percentage value (see Fig.SI.1)
We chose window sizes that provide on average bin size of 5 data points or more. For window sizes that have the same average bin size, we chose the smallest and biggest percentage of points in the window. The resulting window sizes to study are:

```
[ 5,  6,  7,  8,  9, 10, 11, 12, 13, 14, 15, 16, 17, 19, 20, 21,
    24, 25, 30, 33, 34, 40, 49]
```

We then construct the windows with these sizes with an overlap ranging between $[5\%, 85\%]$ of the window size.

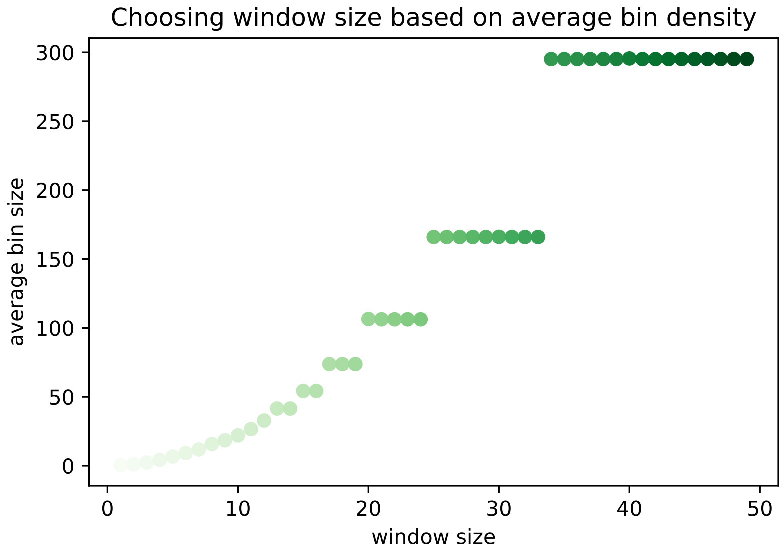

Figure SI.1: (Colour online) **Average bin size as window size varies.**

Finding the best combination of window size and overlap

We study 368 pairs of window size and overlap using general structural parameters of the resulting networks. In particular we take into consideration connected components and edge density, the ratio between existing edges and possible edges.
Here are the limitations that we considered:
STEP 1 - limiting the number of connected components: we impose to have more than 1 connected component that is not and isolated point - top plot and at least 50% of nodes in the graph to be isolated - bottom plot

STEP 2 - we limit the edge density of the connected components to 1. To do so, we assume the number of nodes in the lattice to be square and we can always consider that N_c, the number of nodes in a connected component, is $n^2 \leq N_c < (n+1)^2$. We chose to maintain the same inequality in the edge densities $\frac{E_c}{L_n} \geq 1 \geq \frac{E_c}{L_{n+1}}$ where E_c is the number of edges in the connected component c , and L_n is the number of edges in the square lattice with n nodes on each side.
After having imposed these two rules we are left with a few optimal parameters for our analysis.

full (window size, overlap) $= [(5, 25), (5, 30), (5, 35), (6, 20)]$

fMRI correlated (window size, overlap) =
$[(5, 25), (5, 30), (5, 35), (5, 40), (6, 20), (6, 25), (7, 20)]$

dopamine (window size, overlap) $= [(5, 25), (6, 20)]$

2 Defining the different regimes for comparison with existing results

For a more detailed comparison of the resulting Mapper network connectivity with existing results in the literature, we decided to divide the links found by the Mapper in 4 different regimes. The regimes were defined to highlight the relation between high/low connectivity in the Mapper with high/low values in the comparing matrices (the differential expression matrix in Fig.3 and the functional connectivity matrix in Fig. 6). In the figures SI.2 and SI.3 we show how the elements in the agreement matrix were divided in the 4 regimes respectively for the whole genome expression, and for the 136 genes identified in Richiardi et al. [2015].

Results for the shortest path distance from ventral tegmental area

In Fig. SI.4 we show the distribution of shortest path distance from the substantia nigra and ventral tegmental area. The distributions represent the path distance from all samples in the seed area to other ROIs present in the same component. ROIs with fewer samples or that are densely connected in the network have a narrower distribution. Overall the results for the two seeds are very similar to each other. This similarity is likely due to the samples from these two areas being clustered together in the same nodes in the network.

3 fMRI data acquisition and pre-processing

Subjects were scanned in a Siemens Connectome Skyra 3T scanner with a Gradient echo echo-planar imaging sequence (TR = 720 ms, TE = 33.1 ms, Kip angle = 52 degrees, multiband factor = 8, slice thickness = 2 mm, 72 slices and 1200 volumes) for the duration of 15 minutes. During the scan, the subjects fixed their gaze on a cross-hair on a black

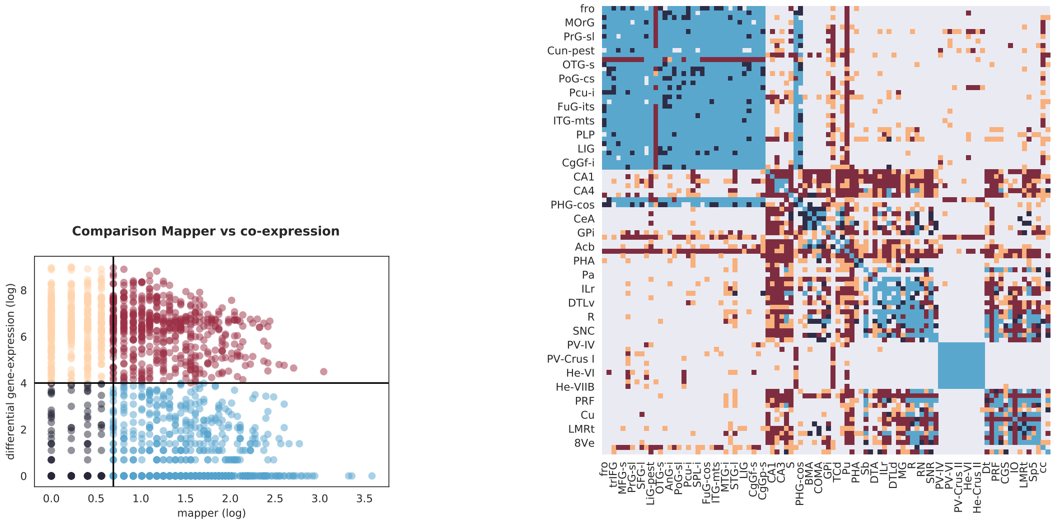

Figure SI.2: (Color online) **Defining the connectivity regimes for whole transcriptome comparison**

(left) Scatterplot depicting the subdivision of the links of the Mapper in the 4 different regimes. On the x axis the values of the agreement matrix divided between high/low connectivity at 2, on the y axis the differential expression of the links divided between high/low at 4, the value at which the difference between the distribution of differential expression in the present and excluded link changes sign Fig. 3d). (right) agreement matrix colored according to the 4 regimes to which each link belongs to.

background. The images were subsequently processed in the following way. First, the structural pipeline was applied to create an undistorted native structural volume space for each subject. T1- and T2-weighted images were aligned, and bias field correction carried out. The pre-processed T1w were used for all subjects. Each subject's native space was then registered to the MNI-152 2-mm space. The next step of segmentation was carried out in Freesurfer. The final part of structural pre-processing generated the NIFTI volume file, to which the functional data was mapped in the next step. This included functional pre-processing to remove spatial distortion, and realignment and registration to the structural MRI, bias field reduction, and normalization of the volumetric 4-D image to a global mean. Finally the 4-D data was masked with a final brain mask derived from a structural NIFTI file. No slice-timing correction was carried out, as the multiband factor required a large number of slices to be acquired close together. The ICA-FIX approach was then applied [Salimi-Khorshidi et al., 2014]. Motion correction was the next step of clean-up, using 24 confound time-series derived from motion estimation of the six rigid-body transformations, and their backwards-looking temporal derivatives. The resultant 12 regressors were squared. The motion parameters are subject to high-pass temporal filtering, and are then regressed out of the data.

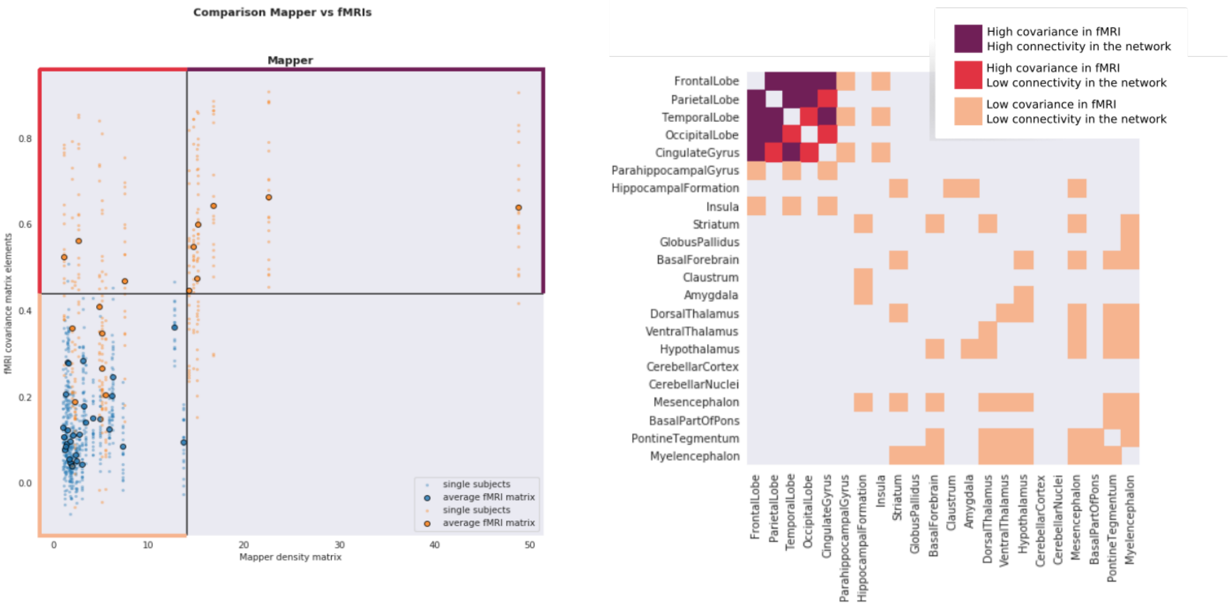

Figure SI.3: (Color online) **Defining connectivity regimes for fMRI comparison** (left) Scatterplot depicting the subdivision of the links of the Mapper in the 3 different regimes. On the x axis the values of the agreement matrix divided between high/low connectivity at 14, on the y axis the functional connectivity of the links divided between high/low at .44, the minimum functional connectivity value within the cerebral cortex (lobes and cyngulate gyrus). (right) agreement matrix colored according to the 4 regimes to which each link belongs to.

4 List of 136 genes used for the fMRI comparison

['ADAM23', 'ANKRD6', 'ATP6V1C2', 'BAIAP3', 'C3orf55', 'CARTPT', 'CCDC39', 'CD70', 'CDC2', 'CNTN6', 'CRYBA2', 'CTXN3', 'CXXC11', 'DMRT3', 'EPN3', 'FEZF1', 'FZD7', 'GAL', 'GLRA3', 'GNA14', 'GNGT2', 'GRP', 'HSD11B1', 'KANK4', 'KCNA1', 'KCNA3', 'KCNA5', 'KCNC1', 'KCTD15', 'KRT1', 'KRT31', 'LAIR2', 'LINC00238', 'LMOD3', 'LRRC38', 'LYPLA2', 'MGP', 'MYH7', 'MYLK3', 'NEB', 'NECAB2', 'NEFH', 'NEUROD6', 'NGFR', 'NOL4', 'NOV', 'NRP1', 'ONECUT2', 'PCP4', 'PIRT', 'PNMT', 'PRR15', 'PRSS23', 'PTGS1', 'RBP4', 'RBPMS2', 'RHOBTB2', 'RSPH9', 'SCARA5', 'SCN1B', 'SCN4B', 'SEMA7A', 'SHD', 'AC092324.1', 'SLC16A6', 'SLC22A10', 'SLN', 'SV2C', 'SYT10', 'SYT2', 'TDO2', 'TGFBI', 'PLAC2', 'TLX2', 'TNNT2', 'TRIM29', 'TSHZ3', 'AMDHD1', 'ASGR2', 'CD163L1', 'COL5A2', 'CYP2C18', 'FAM163A', 'GABRA5', 'GALP', 'GPR26', 'GPR88', 'GPX3', 'HPCAL1', 'IL13RA2', 'ISCU', 'NEXN', 'NKAIN4', 'NPBWR2', 'ONECUT3', 'OR51E2', 'PLCH1', 'PVALB', 'SNAP25', 'SPHKAP', 'TMEM52', 'TSPAN8', 'ZCCHC18', 'ALOX12', 'CALB1', 'CCBE1', 'CD6', 'CDR2L', 'CPLX1', 'ENPP6', 'GMPR', 'GOLT1A', 'GPR20', 'HOXD1', 'HPCA', 'IL33', 'IQCJ', 'KLK1', 'KLK8', 'LGR6', 'TCL1A', 'MS4A8B', 'MYBPC1', 'RP4-725G10.1', 'PYDC1', 'RTP1', 'SEMA3C', 'SH3RF2',

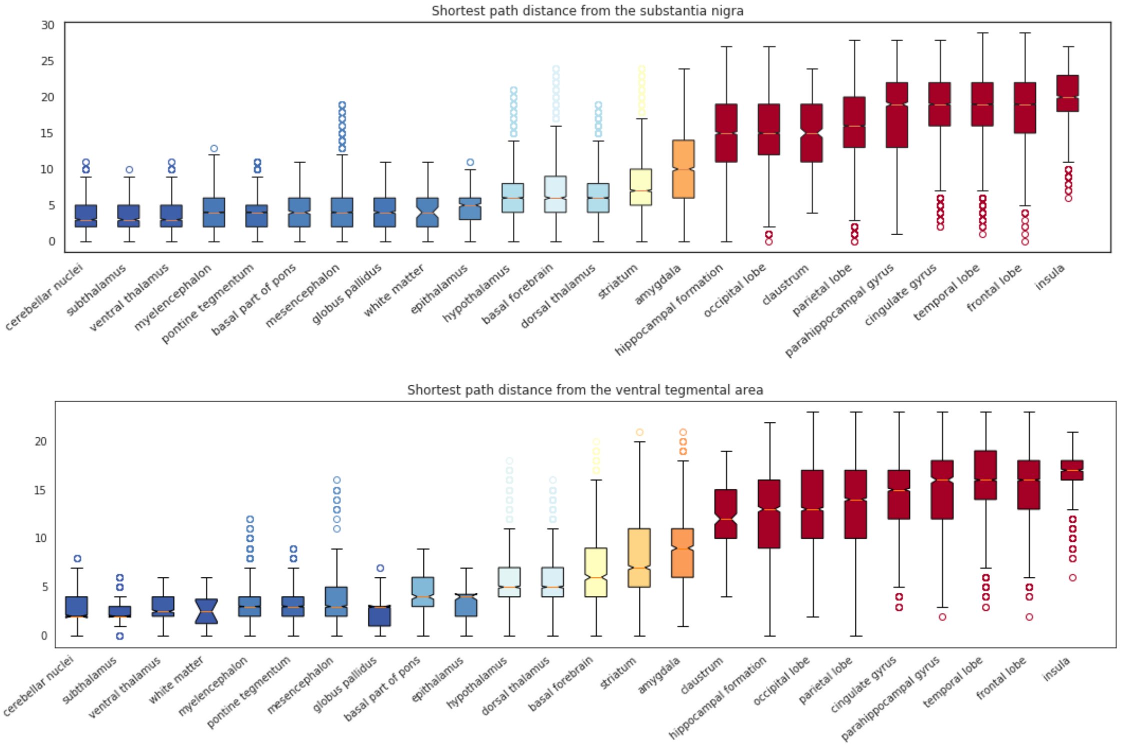

Figure SI.4: (Colour online) **Comparison of the distributions of shortest path distance with seeds in VTA and Substantia nigra**
For each network we identified the nodes in the network containing samples from the ventral tegmental area or substantia nigra and computed the shortest path distance from these nodes to the rest of the network. Here we show the distribution of path distance values for each ROIs ordered (left-right) according to their median value from closest to farthest, for seeds in the substantia nigra (top) and ventral tegmental area (bottom).

'SLC16A5', 'SLC20A2', 'SLC39A12', 'SOST', 'WISP1', 'WISP2', 'WNT4']

5 List of 56 genes used for study on dopamine system

['ADCY2', 'ADCY7', 'CDK5', 'CLIC6', 'COMT', 'DBH', 'DDC', 'DRD1', 'DRD2', 'DRD3', 'DRD4', 'DRD5', 'EPB41', 'EPB41L1', 'EPB41L2', 'EPB41L3', 'FLNA', 'GNAI1', 'GNAI2', 'GNAI3', 'GNAZ', 'GNB1', 'GNB2', 'GNB3', 'GNB4', 'GNG11', 'GNG3', 'GNG4', 'GNG8', 'KCNK3', 'KCNK9', 'MAOA', 'MAOB', 'NAAA', 'OC90', 'PPP1CA', 'PPP1CC', 'PPP1R1B', 'PRKACA', 'PRKACB', 'PRKACG', 'PRKAR2A', 'PRKAR2B', 'PRKX', 'PRKY', 'SLC18A2', 'SLC6A3', 'SNAP23', 'SNAP25', 'SNAP29', 'STX3', 'TH', 'VAMP1', 'VAMP2', 'VAMP3', 'VAMP8']

6 List of functional Networks used in work by Richiardi et al.

['Auditory', 'dDMN', 'high_Visual', 'Language', 'LECN','post_Salience', 'Precuneus', 'prim_Visual', 'RECN', 'Salience','Sensorimotor', 'vDMN', 'Visuospatial', 'Z_restOfBrain']

References

Ricardo JGB Campello, Davoud Moulavi, Arthur Zimek, and Jörg Sander. Hierarchical density estimates for data clustering, visualization, and outlier detection. *ACM Transactions on Knowledge Discovery from Data (TKDD)*, 10(1):5, 2015.

Jonas Richiardi, Andre Altmann, Anna-Clare Milazzo, Catie Chang, M Mallar Chakravarty, Tobias Banaschewski, Gareth J Barker, Arun LW Bokde, Uli Bromberg, Christian Büchel, et al. Correlated gene expression supports synchronous activity in brain networks. *Science*, 348(6240):1241–1244, 2015.

G. Salimi-Khorshidi, G. Douaud, C. F. Beckmann, M. F. Glasser, L. Griffanti, and S. M. Smith. Automatic denoising of functional MRI data: combining independent component analysis and hierarchical fusion of classifiers. *Neuroimage*, 90:449–468, Apr 2014.
